# Ionic and Polyampholyte *N*-Isopropylacrylamide-Based Hydrogels Prepared in the Presence of Imprinting Ligands: Stimuli-Responsiveness and Adsorption/Release Properties

**DOI:** 10.3390/jfb2040373

**Published:** 2011-12-15

**Authors:** Miguel A. Lago, Valerij Ya. Grinberg, Tatiana V. Burova, Angel Concheiro, Carmen Alvarez-Lorenzo

**Affiliations:** 1 Departamento de Farmacia y Tecnología Farmacéutica, Facultad de Farmacia, Universidad de Santiago de Compostela, Santiago de Compostela 15782, Spain;E-Mails: miguelangel.lago@rai.usc.es (M.A.L.); angel.concheiro@usc.es (A.C.); 2 N.M. Emanuel Institute of Biochemical Physics, Russian Academy of Sciences, Vavilov St. 28, Moscow 119991, Russia; E-Mail: grinberg@ineos.ac.ru; 3 A.N. Nesmeyanov Institute of Organoelement Compounds, Russian Academy of Sciences, Vavilov St. 28, Moscow 119991, Russia; E-Mail: burova@ineos.ac.ru

**Keywords:** stimulus-responsive network, imprinted hydrogel, ionic and polyampholite hydrogels, propranolol, ibuprofen, controlled release

## Abstract

The conformation of the imprinted pockets in stimulus-responsive networks can be notably altered when the stimulus causes a volume phase transition. Such a tunable affinity for the template molecule finds interesting applications in the biomedical and drug delivery fields. Nevertheless, the effect that the binding of the template causes on the stimuli-responsiveness of the network has barely been evaluated. In this work, the effect of two ionic drugs used as templates, namely propranolol hydrochloride and ibuprofen sodium, on the responsiveness of *N*-isopropylacrylamide-based hydrogels copolymerized with acrylic acid (AAc) and *N*-(3-aminopropyl) methacrylamide (APMA) and on their ability to rebind and to control the release of the template was evaluated. The degree of swelling and, in some cases, energetics (HS-DSC) of the transitions were monitored as a function of temperature, pH, and concentration of drug. Marked decrease in the transition temperature of the hydrogels, accompanied by notable changes in the transition width, was observed in physiological NaCl solutions and after the binding of the drug molecules, which reveals relevant changes in the domain structure of the hydrogels as the charged groups are shielded. The ability of the hydrogels to rebind propranolol or ibuprofen was quantified at both 4 and 37 °C and at two different drug concentrations, in the range of those that cause major changes in the network structure. Noticeable differences between hydrogels bearing AAc or APMA and between imprinted and non-imprinted networks were also observed during the release tests in NaCl solutions of various concentrations. Overall, the results obtained evidence the remarkable effect of the template molecules on the responsiveness of intelligent imprinted hydrogels.

## 1. Introduction

In contrast to common imprinted networks in which the conformation of the imprinted cavity is rigidly fixed in the 3D-space of the polymer, stimuli responsive networks may undergo remarkable changes in the conformation of their imprinted pockets [1,2,3,4]. Such responsiveness enables to switch on/off the affinity of the networks for the imprinted molecules when tiny changes in pH, temperature, ionic strength, light or concentration of certain substances occur [5,6,7,8]. These features make stimuli-responsive imprinted networks particularly attractive for biomedical purposes and, especially, for site specific drug release. The stimuli-responsive imprinted networks are usually made of a main monomer, which allows the gel to swell and shrink reversibly in response to environmental changes, and some functional monomers able to capture target molecules via multiple-point interactions [1,9,10,11]. Successful conformational imprinting effect is only achieved if the functional monomers develop affinity for the template when they come close to each other, forming the imprinted pocket (receptor), but when they are separated, the affinity diminishes [2,12]. Thus, the polymerization conditions should be chosen to obtain the polymer network at the collapsed state in the lowest energy conformation, in order to create the driving force to memorize the conformation of the network and the location of functional groups that form the imprinted pockets. Removal of the template molecules after polymerization, as well as the stimuli that make the network swell, may destroy the initial conformation. In the presence of the template molecules (rebinding) the memory of the imprinted conformation is recalled by an induced fit or when the suitable stimulus causes the network to shrink. So far, the ability to swell/shrink has been shown useful to modulate the amount of drug loaded and the release rate from stimuli-responsive imprinted networks [6,7,8,10,11,13,14,15,16,17,18,19]. From a theoretical point of view, the study of responsive imprinted gels has contributed to a better understanding of the memorization of conformations in heteropolymer networks and to develop protein-mimicked systems endowed with recognition features [11,14].

A relevant aspect that has received much less attention refers to the effect that causes the binding of the template molecules on the stimuli-responsiveness of the network. It has been previously observed for non-imprinted networks (*i.e.*, synthesized in the absence of template molecules) that copolymerization of N-isopropylacrylamide (NIPA) with ionic monomers notably shifts the critical temperature from 32 °C to remarkably higher values [20,21,22,23]. In the case of polyampholyte networks, Coulombic attraction between oppositely charged mers (salt bridges) alters the network conformation and usually leads to lower increments in the critical temperature [24]. Such internal complex may hinder to certain extent the recognition of a target substance by one of the ionic monomers. On the other hand, the oppositely charged monomer that becomes free as the template is bound progressively increases the network's ionic character, and thus the temperature-responsiveness becomes altered. The aim of our work was to elucidate the influence of ionic templates on the responsiveness of NIPA-based hydrogels and on their ability to rebind and to control the release of the template. Namely, the hydrogels were synthesized using acrylic acid (AAc) and *N*-(3-aminopropyl) methacrylamide (APMA) as functional monomers, and the β-agonist drug propranolol (weak base) and the non-steroidal anti-inflammatory drug ibuprofen (weak acid) as templates. The responsiveness to pH, temperature and ionic strength, and particularly the changes that the presence of the drug molecules causes in the stimuli-sensitiveness were characterized in detail by means of swelling measurements and high-sensitivity differential scanning calorimetry (HS-DSC) [22]. Then, imprinted and non-imprinted networks were compared regarding the uptake of the drugs and the suitability for achieving a sustained release. The information obtained may help to the rational design of drug delivery systems and drug-eluting combination products based on stimuli-responsive imprinted networks.

## 2. Experimental Section

### 2.1. Materials

*N,N′*-methylenebis(acrylamide) (BIS) was obtained from Bio-Rad Laboratories, USA. Acrylic acid (AAc), ibuprofen, *N,N,N′,N′*-tetramethylethylenediamine (TEMED), ammonium persulphate (APS) and 2,2′-azobis(isobutyronitrile) (AIBN) were from Sigma Aldrich, Spain. *N*-(3-aminopropyl) methacrylamide (APMA) was from Polysciences Europe (Eppelheim, Germany). *N*-isopropylacrylamide (NIPA) was provided by Kohjin Co. Ltd., Japan. Ibuprofen sodium and propranolol hydrochloride were from Fagron (Valencia, Spain). Water purified by reverse osmosis (resistivity > 18.2 MΩ·cm; MilliQ^®^, Millipore: Madrid, Spain). All other reagents were of analytical grade.

### 2.2. Hydrogel Synthesis in DMSO

Gels were prepared by free radical polymerization using NIPA (6 M) and cross-linker BIS (40 mM) in dimethyl sulfoxide (DMSO, 50 mL). Some hydrogels also contained APMA (120 mM) and/or AAc (120 mM). To evaluate the effect of polymerization in the presence of templates on hydrogels features, ibuprofen (120 mM) or propranolol (120 mM) were added to some monomers solutions. After the addition of AIBN (10 mM, initiator), the solutions were immediately injected into molds constituted by two glass plates (10 × 10 cm) separated by a silicon frame 2.0 mm wide. Polymerization was carried out at 70 °C for 24 h. Then, the gels were taken out of the molds, cut as discs (10.8 mm in diameter) and consecutively washed with deionized water (four days), pH 7.4 phosphate buffer (one day), 100 mM NaCl (one day), 10 mM HCl (one day), and again with deionized water (eight days). The gels were removed from the solution by means of tweezers and dried under vacuum at 40 °C for 1 week.

### 2.3. Swelling Cycles at 4 and 37 °C

Dried hydrogel discs were placed in vials containing water at 4 or 37 °C and let to equilibrate for 40 h. Then the vials were cyclically transferred to a thermostated bath at 37 °C and to the fridge at 4 °C. The weight and the diameter of the discs were recorded 15 and 45 min after the change in temperature. The degree of swelling was estimated as:

(1) $$\text{Q}\left(\%\right)={\left({\text{W}}_{\text{t}}-{\text{W}}_{0}\right)/\text{W}}_{0}\cdot 100$$

where W_t_ and W_0_ represent the weight of the hydrogels at the beginning of the test and at a time *t* during the test. All experiments were carried out in triplicate.

### 2.4. Energetics of Phase Transition

The energetics of volume phase transition was studied by high-sensitivity differential scanning calorimetry (HS-DSC) [22,25,26]. Hydrogel samples were preliminarily immersed in water and broken down to obtain an average particle size of about 10 μm using a Downs glass homogenizer of biological tissues. The suspensions were diluted half-and-half by stock buffer solutions with various NaCl and template concentrations. The final PNIPA (poly-*N*-isopropylacrylamide) concentration in the sample dispersion was around 1–3 mg·mL^−1^. The diluted suspensions were kept overnight at 4 °C under careful stirring and used for calorimetric measurements. The calorimetric measurements were carried out with a differential adiabatic scanning microcalorimeter DASM-4 (Biopribor, Pushchino, Russia) within temperature range 10–70 °C at a heating rate of 1 K·min^−1^ and excess pressure of 2.5 bar. As a rule, two successive scans were performed. Results of the second scan were used for calculation of the apparent partial heat capacity of PNIPA polymer network. The peak temperature of the heat capacity curve was assumed as the transition temperature, *T_t_*. The transition heat capacity increment, *Δ_t_C_p_*, was determined as the difference between partial heat capacities of the network in collapsed and swollen states at the transition temperature. Base line of the phase transition was approximated by a cubic polynomial. After its subtraction the excess heat capacity function of transition was obtained. The transition enthalpy, *Δ_t_H*, and the transition width, *Δ_t_T*, were determined as the area under this curve and as the width of the curve at its half-height, respectively. Both *Δ_t_Cp* and *Δ_t_H* parameters were normalized per gram of PNIPA present in the hydrogel. The program Nairta (Institute of Biochemical Physics, Moscow) was used for data processing and calculations of the phase transition parameters.

### 2.5. Drug Loading

Discs of each dried hydrogel were immersed in ibuprofen sodium (1.0 or 4.75 mM) or propranolol (0.1 or 1.0 mM) aqueous solution (pH 6.9) at 4 °C (three replicates) or at 37 °C (three replicates) and then kept protected from light for one week. Ibuprofen and propranolol concentration was spectrophotometrically monitored at 273 and 289 nm, respectively (Agilent 8453, Germany). The amount of drug loaded by each disc was then calculated as the difference between the initial and the final quantities of drug in the medium. The drug-loaded hydrogel discs were removed from the medium and dried in an oven at 40 °C until equilibrium.

### 2.6. Drug Release

Dried drug-loaded hydrogel discs were immersed in 5 mL of water at 37 °C for 2 h. Then, the medium was replaced by 5 mL of 0.009, 0.09 or 0.9% NaCl aq. solution at 37 °C. The amounts of drug released were determined spectrophotometrically in samples of medium, which were again returned to each vessel so that the liquid volume was kept constant. After 48 h, the discs placed in 0.009 or 0.09% NaCl solution were transferred to 0.9% NaCl solution.

## 3. Results and Discussion

### 3.1. Hydrogel Synthesis and Temperature-Responsive Swelling

The ionic monomers and the drugs readily dissolved in DMSO and mixed with the other components of the hydrogels. After polymerization, transparent and consistent networks were obtained. An intensive washing step was then carried out in order to remove DMSO, unreacted substances and the drug molecules used as templates during synthesis; the cleaning was monitored by recording the absorbance of the washing solutions.

**Figure 1:**
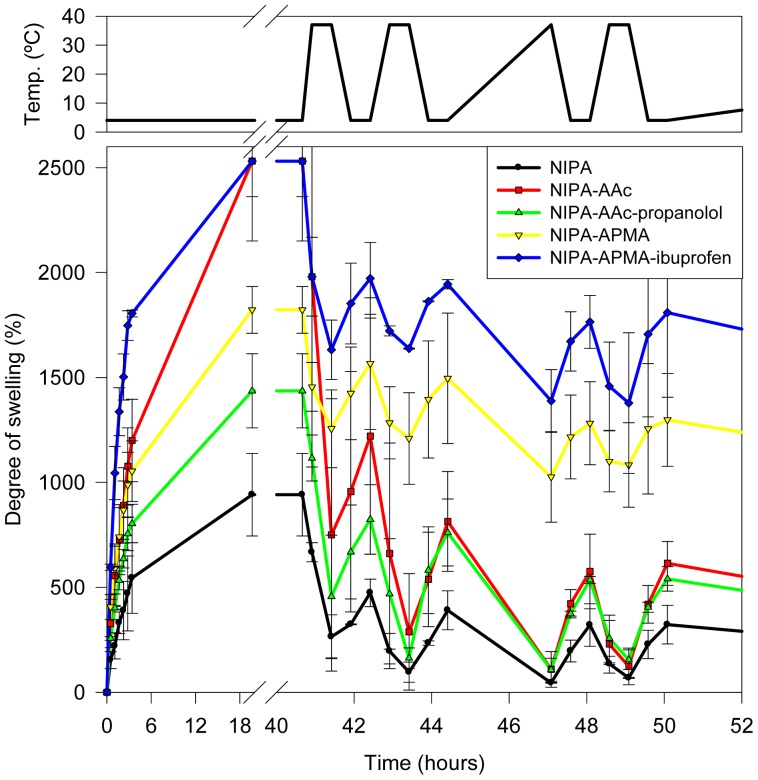
Temperature-responsiveness of the swelling of networks initially placed in cold water (4 °C) and that underwent cyclic changes in temperature in the 4–37 °C range.

It is well known that copolymerization of NIPA with ionic monomers can alter the temperature threshold that induces the phase transition [21,22]. Thus, the first step was to test if the clean networks evidenced temperature-responsiveness in water in the common range of temperatures of biomedical applications, *i.e.*, from 4 °C for storage of labile substances to 37 °C for mimicking the physiological conditions. Two sets of experiments were run in parallel: some dried hydrogels were immersed first in cold water (4 °C) while other set was initially placed in warm water (37 °C). Figure 1 shows the changes in degree of swelling of the first set of hydrogels. At 4 °C (much below the LCST, lower critical solubility temperature) all networks behave as superabsorbent. Nevertheless, a remarkable influence of the comonomer and the template drug added during synthesis was noticed. The swelling at 4 °C ranked in the order: NIPA < NIPA-AAc-Propranolol < NIPA-APMA < NIPA-AAc < NIPA-APMA-Ibuprofen. Polyampholyte NIPA-APMA-AAc (data not shown) behaved as NIPA network, which indicates that internal cationic/anionic pair formation (salt bridges) occurred and that both types of monomers have been incorporated in a similar proportion. The presence of propranolol and ibuprofen in the polymerization mixture caused different effects in the swelling degree. It has been reported that cationic drugs can strongly interact with AAc [27]. In the monomeric solutions without propranolol, AAc tends to interact with neighbour AAc through hydrogen bonds. Propranolol-AAc complexes can prevent the formation of AAc clusters and thus each AAc-mer might be more separate each other when polymerized in the presence of propranolol. Thus, when the drug is removed during the washing step, the repulsions among ionized AAc may be less intense than if AAc were closer in the space. As a consequence, the degree of swelling is also lower. Ibuprofen-APMA interactions are expected to be weaker in the polymerization mixture since the monomer was used in its salt form, namely hydrochloride, and the drug is also protonated. Thus, synthesis in the presence of ibuprofen may cause a different rearrangement of APMA monomers than when no drug is present.

Once fully swollen at 4 °C, the hydrogels were cyclically heated (37 °C) and cooled down (4 °C) to evaluate the temperature-sensitiveness and the rate and reproducibility of the volume phase transitions (Figure 1). At 37 °C, a fast shrinking was observed for all hydrogels. Re-swelling also occurred rapidly when transferred to 4 °C medium. Those hydrogels prepared with NIPA solely or plus AAc with or without propranolol almost reached the collapse in less than 15 min at 37 °C (swelling below 100%), indicating that the hydrophobic interactions among the isopropyl groups of NIPA are the main responsible for the shrinking of the networks. Hydrogels bearing APMA did not shrink as much as the others in spite of the proportion of AAc and APMA used to synthesize the networks was the same. This finding is explained by the different degree of ionization of both monomers. It is well known that the *pKa* of AAc raises as the monomers get close to each other [20,21] and, as a consequence, only partial ionization of the AAc mers is expected in the networks. Therefore, the contribution to the hydrophilicity of the network at 37 °C is minor. By contrast, APMA may remain as hydrochloride and thus positively charged (*pKa* 8.3 in literature [28] and 10.2 experimentally determined). The presence of the ionized groups in the networks led to swelling values in the shrunken state still above 1000%.

**Figure 2:**
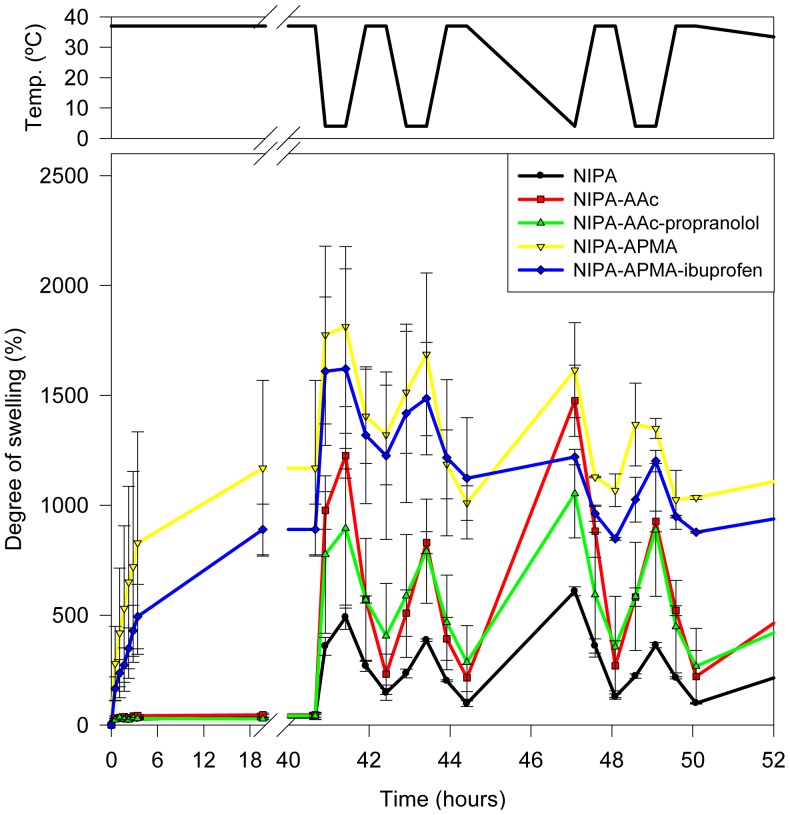
Temperature-responsiveness of the swelling of networks initially placed in water at 37 °C and that underwent cyclic changes in temperature in the 4–37 °C range.

Similar temperature-induced swelling changes were observed when the hydrogels were initially placed in water at 37 °C (Figure 2). Dried hydrogels prepared with NIPA solely or plus AAc with or without propranolol did not increase the volume when placed in the medium at 37 °C. By contrast, hydrogels bearing APMA swelled to ca. 1000%. When the temperature decreased to 4 °C, all networks swelled to a larger extent (Figure 2) following a pattern similar to that shown in Figure 1 after successive changes in temperature. Therefore, the networks behave as intelligent ones with rapid temperature-responsiveness and reproducible values of swelling and shrinking in water during several cycles.

### 3.2. Phase Transition Temperature in Salt and Drug Solutions

**Figure 3:**
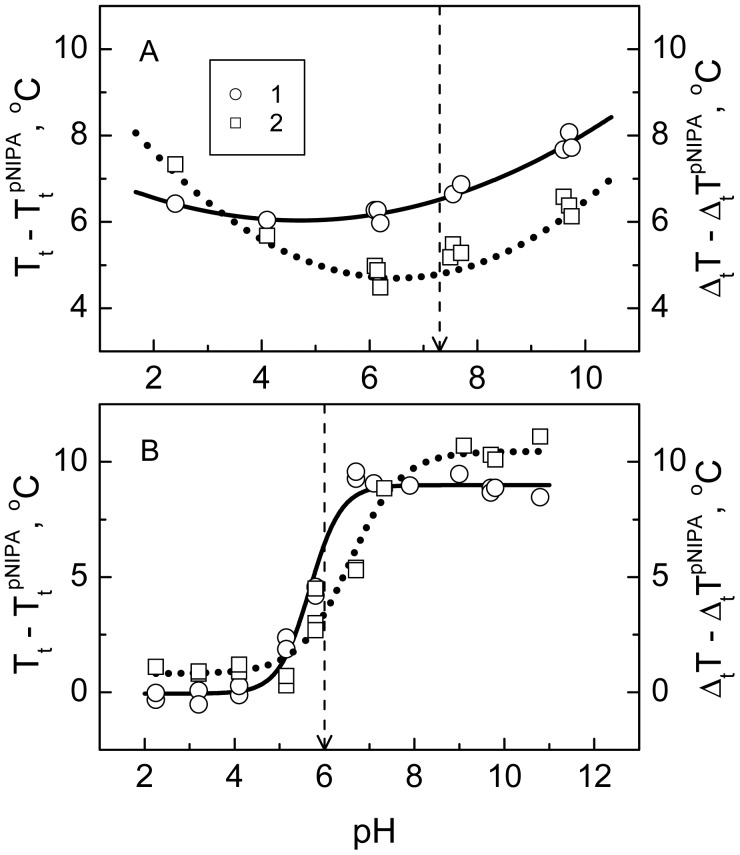
Increments of the transition temperature (1 continuous line, left ordinate axis) and width (2, dotted line, right ordinate axis) *vs.* pH for the polyampholyte *N*-isopropylacrylamide (NIPA)-APMA-AAc (**A**) and the charged NIPA-AAc (**B**) hydrogels. The dashed arrows indicate (**A**) the pI value of the polyampholyte gel calculated from its apparent copolymer composition and the dissociation constants of AAc (*pK_a_* 4.3) [30] and APMA (*pK_a_* 10.2, determined experimentally), and (**B**) the half-dissociation pH value of poly(AAc) [30].

The thermodynamics of the phase transition (namely transition temperature and width) of the networks in media of various pH and salt and template concentrations was evaluated in order to gain an insight into the behavior of the hydrogels in environments that resemble the physiological conditions better than just water. It was of interest to compare some features of the collapse of the polyampholyte NIPA-APMA-AAc gel with those of the charged NIPA-AAc gel that has the same NIPA/AAc ratio and the same cross-linking density. The pH-dependences of the transition temperature and width of the polyampholyte and charged gels are shown in Figure 3. Values of these parameters are calculated relative to the corresponding parameters of the PNIPA hydrogel. The values of the transition parameters of the polyampholyte gel exceed those of the reference PNIPA gel within the whole pH range. In other words, the polyampholyte gel undergoes the transition at the higher temperature and over wider temperature interval as compared with the PNIPA gel of the same cross-linking density. Besides, both transition parameters of the polyampholyte gel pass through a minimum upon changing pH. The minimum is particularly clearly seen in case of the transition width. From a general point of view one can expect that the polyampholyte gel should have an isoelectric point (pI), where the net charge of its network is zero. Upon shifting pH to the left and right from the pI, the network acquires a positive or negative net charge respectively. We have roughly estimated the pI value of the polyampholyte gel in the approximation of the proton binding to the independent sites (groups APMA and AAc) [29], using an apparent copolymer composition of the gel and the values of the dissociation constants (*pK_a_* 10.2 and *pK_a_* 4.3, respectively) [30] of these groups. The value pI 7.3 was obtained. It is marked in Figure 3A by a dashed arrow. It is seen that the minima of the dependences of the transition parameters of the polyampholyte gel are located approximately in the vicinity of the hydrogel pI. It is known that an important factor affecting the collapse energetics and structure of the polyampholyte gels is the formation of salt bridges between the oppositely charged groups of the gel network [24]. A maximal number of such bridges exists in the pI, where the number of the oppositely charged groups is maximal. Thus, it can be concluded that the presence of the salt bridges plays in favor of the collapse of the polyampholyte gel upon heating and makes its structure more cooperative (decreasing temperature and width of the transition at pI). At the same time, when comparing the polyampholyte gel with the reference PNIPA gel we should note that the inclusion of the salt bridges into the PNIPA network impedes its collapse and leads to a definite fragmentation of its structure. It is believed that these effects are related to the frustration phenomenon, *i.e.*, imposition of the restrictions on the subchain conformations in result of the formation of salt bridges between some subchain links [24].

The pH-dependences of the transition parameters of the charged NIPA-AAc gel seem to be similar to the dissociation curve of the polyacrylic acid. These are the sigmoid curves with an inflection point in the vicinity of pH 6 [30]. At pH 2, where the AAc dissociation is essentially suppressed, the transition parameters of the NIPA-AAc gel do not differ notably from the parameters of the reference PNIPA gel. With increasing pH the network of the NIPA-AAc gel acquires a larger charge, the transition temperature increases, and the transition is broadened. These changes are approximately exhausted at pH > 9. A probable cause of these changes in the transition parameters of the charged NIPA-AAc gel is most likely an electrostatic repulsion which impedes the rapprochement of subchains and perturbs the cooperative character of their interaction on the way to the collapsed state.

**Figure 4:**
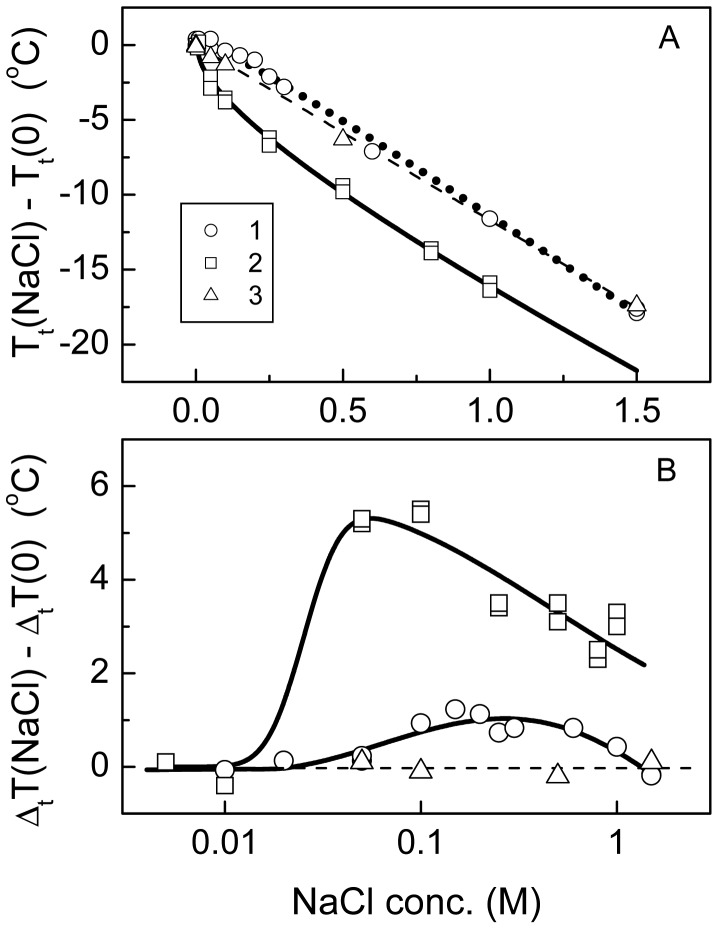
Changes in the transition temperature (**A**) and width (**B**) *vs*. NaCl concentration for the polyampholyte NIPA-APMA-AAc (1), NIPA-AAc (2) and NIPA (3) hydrogels: A—solid and dotted lines correspond to equation (1); dashed line corresponds to equation (2).

A strong effect of sodium chloride on the transition temperature and width of the NIPA-APMA-AAc, NIPA-AAc and NIPA hydrogels was observed (Figure 4). The transition temperature decreased with increasing NaCl concentration (Figure 4A). The dependences of the transition temperature on salt concentration, *T_t_*(*C_s_*), for the polyampholyte and charged gels were curvilinear at the initial sections and then tend to be linear at higher salt concentrations, while that for the neutral NIPA gel was strictly linear over all salt concentration range studied. In the case of the polyampholyte and charged gels, these dependences can be approximated by the equation:

(1) $$T_{t}\left(C_{s}\right)-T_{t}\left(0\right)=K_{e}C_{s}^{0.5}+K_{s}C_{s}$$

while for the NIPA network, the Sechenov type equation should be used:

(2) $$T_{t}\left(C_{s}\right)-T_{t}\left(0\right)=K_{s}C_{s}$$

The parameters *K_e_* and *K_s_* take into account the electrostatics screening and the lyotropic effects on the transition free energy, respectively. The approximation of the data presented in Figure 4A gives *K_e_* = −8.8 ± 0.8 K·M^−0.5^ for the NIPA-AAc hydrogel, and *K_e_* = 3.0 ± 0.5 K·M^−0.5^ for the polyampholyte gel. The negative value of *K_e_* in the case of the NIPA-AAc hydrogel indicates a decrease in the free energy of the collapsed state of the network due to salt screening of electrostatic repulsions. Alternatively, the salt screening of electrostatic interaction among the opposite charges of the network in the case of the polyampholyte gel gives rise to an increase in the free energy of the collapsed state. Thus the *K_e_* parameter of the polyampholyte gel is positive. Note, however, that thescreening effect on *T_t_* is expressed for the polyampholyte gel significantly less than for the chargedgel. It shows probably that salt bridges in the collapsed state of the polyampholyte gel are relatively stable and do not dissociate at low salt concentrations. The lyotropic constants *K_s_* of thepolyampholyte and NIPA-AAc hydrogels (−14.4 ± 0.5 and −7.3 ± 0.9 K·M^−1^, respectively) are rather close to those of the neutral NIPA gel (−11.7 ± 0.1 K·M^−1^). This seems to disclose that for all hydrogels the salt effect on *T_t_* at relatively high salt concentrations is mainly connected with the hydrophobic NIPA component of the gels. The negative value of *K_s_* is a consequence of the preferential increase in the free energy of the swollen state due to the salting-out phenomenon.

The effects of NaCl concentration on the transition width of the polyampholyte and NIPA-AAc hydrogels were more complex (Figure 4B). The dependences of the transition width on NaCl concentration for both gels pass through a maximum, particularly evident for NIPA-AAc hydrogel, while no dependence was recorded for the neutral NIPA gel. It is particularly interesting that the maximum occurred in the range of the physiological salt concentration, namely at or slightly below 0.153 M (*i.e.*, 0.9% NaCl isotonic medium). The transition width is a merit of the system cooperativity: the higher is the system cooperativity, the narrower is the transition. The presence of different domains in a given network leads to the broadening of the transition [22]. Alternatively, the transition must become narrower in result of coalescence of the domains. Thus, the represented dependences of the transition width on salt concentration for the polyampholyte and charged gels indicate changes in their domain structure as NaCl concentration raises. In the case of NIPA-AAc hydrogel, the disintegration into domains at very low salt concentration (less than 0.05 M) could be attributed to an increase in electrostatic repulsion between the gel subchains due to a trivial decrease in the *pK_a_* of carboxyl groups, resulting in the increase in their ionization degree. In the case of the polyampholyte gel, a reason for the apparition of domains at low salt concentrations can be a partial dissociation of the salt bridges. On the other hand, coalescence of domains observed for both gels at relatively high salt concentrations could be caused by salting-out enhancement of hydrophobic interaction of the network subchains.

**Figure 5:**
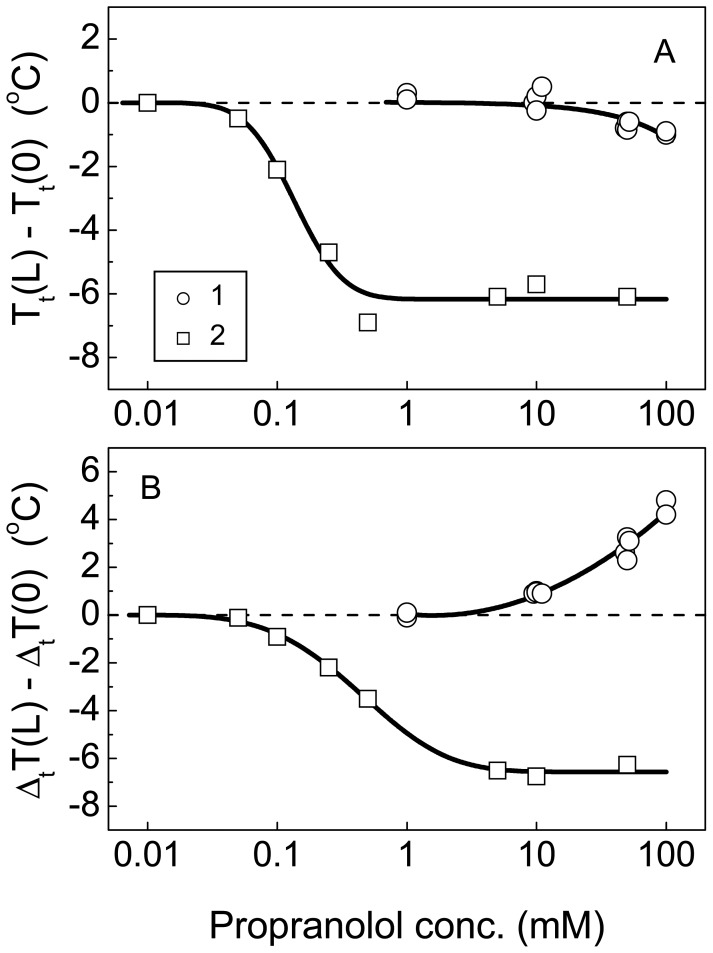
Dependence of the transition temperature (**A**) and width (**B**) of the polyampholyte NIPA-APMA-AAc (1) and charged NIPA-AAc (2) gels on propranolol concentration.

For hydrogels intended to behave as drug delivery systems, it is of relevance to know to what extent the binding of oppositely charged drugs can alter the phase transitions and consequently modify the swelling (and ultimately the mesh size) and drug diffusion through the networks. Influence of the binding of the positively charged amphiphilic ligand, propranolol, on the collapse of the polyampholyte and NIPA-AAc hydrogels is shown in Figure 5. When propranolol concentration increased, the transition temperature of NIPA-AAc hydrogel decreased at first (more than 6 °C) and then reached a constant value (Figure 5A). The transition temperature of the polyampholyte gel tends to decrease only at rather high concentrations of the ligand. Note that the changes in the transition temperature of the polyampholyte gel induced by the drug binding are significantly smaller than those of the charged gel. The different transition behavior of the polyampholyte and the NIPA-AAc hydrogel in the presence of propranolol is particularly evident from the plot of the transition width on ligand concentration (Figure 5B). The transition width of the NIPA-AAc hydrogel diminished with increasing ligand concentration following a pattern that resembled that of the transition temperature. This means that the binding of propranolol and the subsequent neutralization of the AAc charges results in a weakening of the electrostatic repulsion of the network subchains and consequently leads to the coalescence of the gel domains. For the polyampholyte gel, the opposite tendency is observed. The transition width increases upon the increase in the ligand concentration. Propranolol can competitively displace APMA from the interaction with AAc groups, which results in a partial neutralization of negative charges of the network by the drug and the release of free APMA with non-neutralized positive charges. This results in a net positive charge of the network subchains. Repulsion forces arising between the subchains cause apparition of the domains. Thus, the comparison of the transition behavior of the polyampholyte and NIPA-AAc hydrogel discloses significant thermodynamic and structural differences. Mainly, the polyampholyte gel is less sensitive to changes in environmental variables such as pH, salt concentration and the presence of charged ligands. Such a stability of the network structure is a consequence of structural frustrations, that is, internal salt bridges between oppositely charged groups fixing some or other subchain conformations as additional cross-links.

### 3.3. Drug Uptake

**Figure 6:**
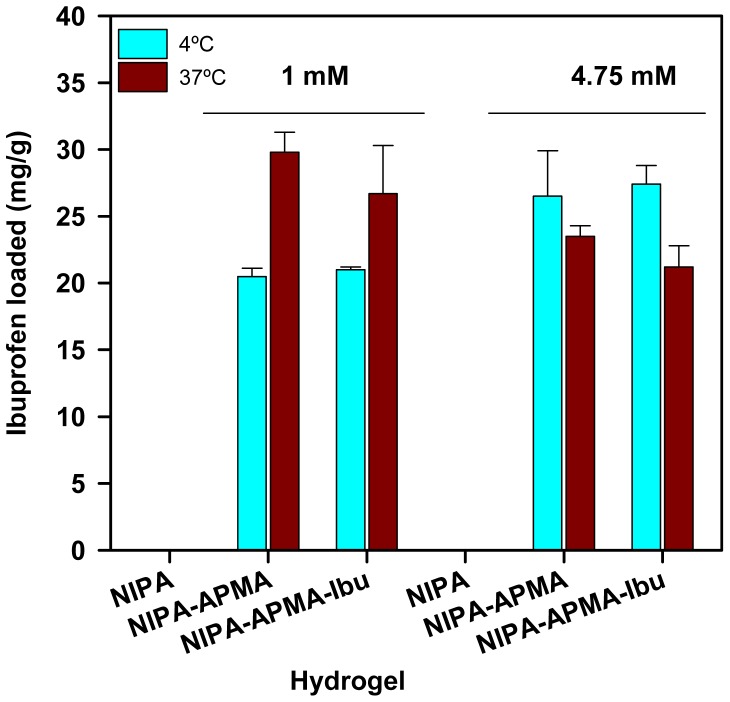
Ibuprofen loaded (mg/g) by NIPA (non-perceptible values) and NIPA-APMA hydrogels when immersed in 1 or 4.75 mM drug aqueous solutions at 4 °C or 37 °C.

**Figure 7:**
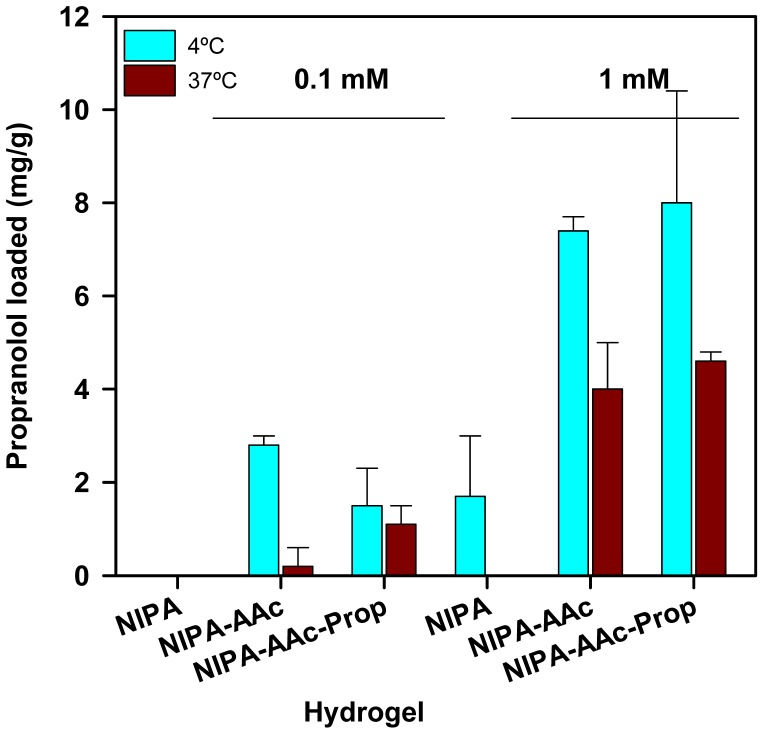
Propranolol loaded (mg/g) by NIPA and NIPA-AAc hydrogels when immersed in 0.1 and 1 mM drug aqueous solutions at 4 °C or 37 °C.

The ability of the hydrogels to uptake propranolol and ibuprofen when immersed in drug aqueous solutions was quantified at both 4 and 37 °C and at two different drug concentrations. Both drugs were tested at 1 mM, which was previously seen by HS-DSC (Figure 5) that caused major changes in the network domains. Propranolol was also tested at lower concentration (0.1 mM) due to solubility limitations, while ibuprofen at higher concentration (4.75 mM) since the therapeutic doses are greater, but not exceeding its critical micellar concentration to avoid interferences due to the molecular aggregation [31]. Drug loading reached the equilibrium in less than 24 h. PNIPA solely hydrogels did not load a significant amount of any drug, while copolymerization with APMA or AAc remarkably enhanced the affinity for ibuprofen (Figure 6) and propranolol (Figure 7).

The amounts of ibuprofen loaded by NIPA-APMA networks were remarkably high and similar for both drug concentrations tested, suggesting saturation of the binding capability already in 1 mM ibuprofen medium. Although minor, effects of temperature on loading in 1 and 4.5 mM drug solutions were observed. The uptake increased with ibuprofen concentration at 4 °C but slightly decreased at 37 °C. This small difference can be attributed to the somewhat smaller degree of swelling at 37 °C (Figure 1 and Figure 2). Nevertheless, at both temperatures NIPA-APMA networks swelled up to a large extent and, therefore, the effect of network mesh size on ibuprofen diffusion should be minor. It is interesting to note that the presence of the ligand during polymerization (codes NIPA-APMA-ibu in Figure 6) led to a lower uptake of ibuprofen at 37 °C and for the two concentrations tested, compared to the network prepared in the absence of the drug (non-imprinted one). A similar effect has been previously found with drug-imprinted networks when loaded in drug solutions that saturate the binding ability and has been attributed to the fact that the drug causes a rearrangement of the functional monomers (APMA in our case) to gather to form binding sites with high affinity for the ligand [32]. As more monomers are involved in the binding of each drug molecules to the imprinted network, less drug molecules are adsorbed. This hypothesis is also supported by the fact that the differences in drug uptake between networks synthesized in the presence and absence of ibuprofen are only noticed when the networks are shrunken, namely in a conformational state similar to that upon synthesis. At 4 °C, the networks are more swollen and the APMA mers become far apart acting each one as a binding site capable to establish electrostatic interactions with one ibuprofen molecule. Thus, the differences between the imprinted and non-imprinted networks are minimized.

In the case of propranolol, the effects of drug concentration and temperature on the amount of drug loaded by NIPA and NIPA-AAc hydrogels were notable (Figure 7). The higher the concentration, the greater the amount loaded. Even NIPA network showed a small uptake when immersed in 1 mM propranolol concentration at 4 °C, which indicates that the drug can establish non-specific interactions with PNIPA chains; however, the collapsed state at 37 °C hinders drug uptake. Copolymerization with AAc notably enhanced drug loading owing to the contribution of the ionic interactions. Increase in drug concentration from 0.1 to 1 mM lead to ca. 3-fold increase in the amount loaded. Nevertheless, the lower amount of propranolol loaded compared to that achieved in the ibuprofen tests suggest that the binding capacity is not fulfilled. Under these conditions, the higher affinity of the networks synthesized in the presence of the drug (imprinted ones) is seen as an increase in the propranolol uptake at 37 °C, namely the collapsed state as upon polymerization. The arrangement of AAc mers to gather to form suitable binding sites for the drug is evidenced when the networks are at the shrunken state and the few drug molecules in the solution (diluted solution) can interact with the most perfectly formed binding sites. Such an affinity overcomes to some extent the hindrance to diffusion caused by the decrease in mesh size at 37 °C. Differences in the degree of swelling between imprinted and non-imprinted networks also corroborate this hypothesis.

### 3.4. Drug Release

**Figure 8:**
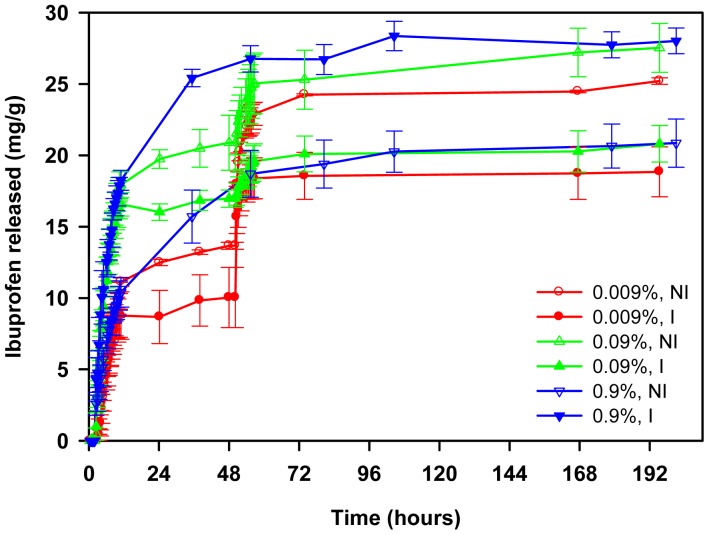
Ibuprofen release profiles in water (first 2 h; no release) and in NaCl solutions (subsequent time) at 37 °C from NIPA-APMA hydrogels loaded in 1 mM ibuprofen solution. After 48 h, the 0.009 or 0.09% NaCl solutions were replaced by 0.9% NaCl. Hydrogels synthesized in the presence of ibuprofen (imprinted) are indicated as “I”.

**Figure 9:**
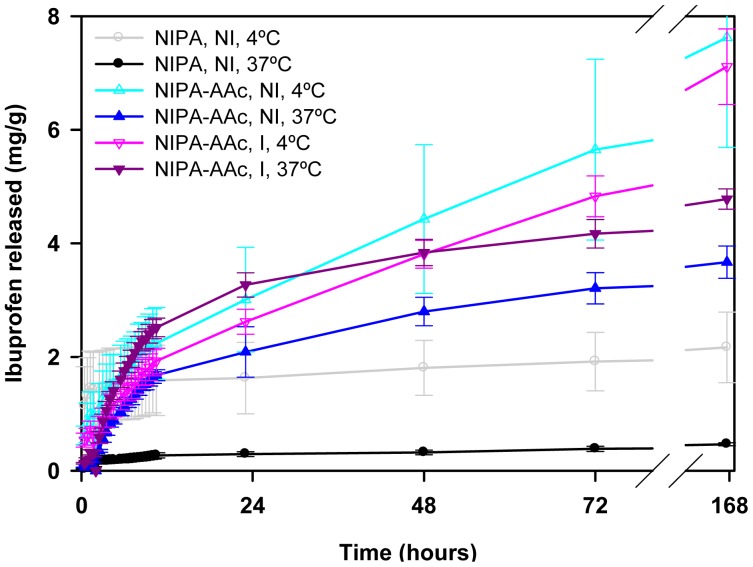
Propranolol release profiles in water (first 2 h) and in NaCl solutions (subsequent time) at 37 °C from NIPA-AAc hydrogels previously loaded by immersion in 1 mM propranolol solution at 4 or 37 °C. Hydrogels synthesized in the presence of propranolol (imprinted) are indicated as “I”.

Release tests were carried out in water for the first two hours and, then, the hydrogels were transferred to NaCl solutions of various concentrations, namely 0.009, 0.09 or 0.9%. In this way, information about the role of the ionic interactions can be obtained and the performance of the hydrogels under physiological mimicking conditions can be elucidated. NIPA-APMA networks did not release ibuprofen at all when immersed in water for 2 h (Figure 8), highlighting that the ionic interactions are so strong that in the absence of competitive ions no release occurs. In the case of propranolol, only NIPA network showed a burst release (100% released in few minutes) due to weak, non-specific interactions with the drug, while NIPA-AAc networks just led to a minor release (Figure 9). Replacement of water for saline medium triggered the release and a marked effect of NaCl concentration on both amount released and release rate was observed.

**Figure 10:**
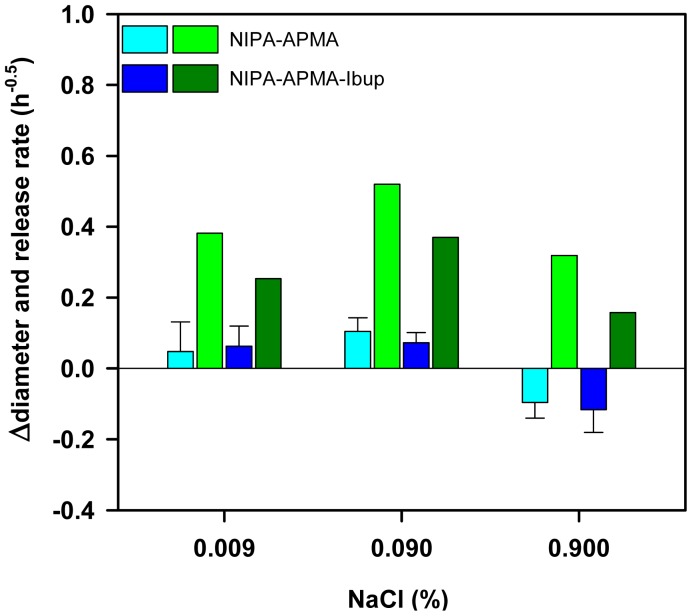
Changes in diameter (blue bars) underwent when non-imprinted (clear colors) and imprinted (dark colors) NIPA-APMA hydrogels loaded with ibuprofen were immersed in NaCl solutions of various concentrations at 37 °C and drug release rate recorded for ibuprofen (green bars).

Drug release profiles fitted well to the Higuchi's equation (R^2^ > 0.98):

(3) $$\frac{M_{t}}{M_{\infty }}=k_{H}\cdot t^{0.5}$$

where M_t_ and M_∞_ represent the amount of drug released at time *t* and the total amount loaded, respectively. The values of the release rate *k_H_* are shown in Figure 10. A detailed analysis of the release process in media of NaCl concentration ranging from 0.009% to 0.9% revealed that non-imprinted NIPA-APMA hydrogels released ibuprofen faster when the ionic strength was intermediate (namely, 0.09%). NaCl is needed to break the template-polymer ionic interactions, favoring the release of the drug, but at the same time the salt causes a salting out effect and makes the network to shrink (Figure 10) and, consequently, drug diffusion becomes hindered [33,34]. Therefore, equilibrium between both effects should result in the maximum release rate. Interestingly, the imprinted networks provided lower release rates of ibuprofen at any NaCl concentration, which confirms that the affinity of the drug for the imprinted pockets is higher than for the non-imprinted network. In 0.09% NaCl the release from the imprinted networks could be triggered but it stopped after a few ibuprofen molecules were released.

## 4. Conclusions

Copolymerization of NIPA with relatively low proportions of APMA or AAc led to hydrogels with ability to uptake oppositely charged drugs, such as ibuprofen and propranolol. The functional monomers notably modify the degree of swelling of the networks at 37 °C and make them to be responsive to pH, ionic strength and drug concentration. Marked changes in the swelling and the transition energetics (temperature and width) were recorded as a function of the drug concentration due to the effect on the effective charge of the polymer subchains. Differences in drug uptake between imprinted and non-imprinted hydrogels were particularly noticeable when the rebinding was carried out in diluted drug solutions at 37 °C. Once loaded, the ionic interactions between the drug and the oppositely charged hydrogels can be only broken in a saline medium with ions able to compete with the drug for the binding sites. Nevertheless, even at physiological 0.9% NaCl medium, sustained release was achieved for several hours due to the shrunken structure of the networks. The most relevant finding of the present work was the drop in several degrees of the transition temperature observed for the ionic NIPA hydrogels after drug loading. This finding can be advantageous to design temperature-responsive networks with a relatively high proportion of ionic monomers and thus with many binding sites for drug hosting. Although, in the absence of the drug the network, it may be not collapse at 37 °C, once loaded with the drug, the temperature responsiveness could be in the physiological range. Further studies about how the network swelling during drug release affects the overall release rate are being carried out in order to evaluate the practical repercussions of these events.

## References

[1] Alvarez-Lorenzo C., Guney O., Oya T., Sakai Y., Kobayashi M., Enoki T., Takeoka Y., Ishibashi T., Kuroda K., Tanaka K., Wang G., Grosberg A.Yu., Masamune S., Tanaka T. (2000). Polymer gels that memorize elements of molecular conformation. Macromolecules.

[2] Ito K., Chuang J., Alvarez-Lorenzo C., Watanabe T., Ando N., Grosberg A.Yu. (2003). Multiple point adsorption in a heteropolymer gel and the Tanaka approach to imprinting: Experiment and theory. Prog. Polym. Sci..

[3] Alvarez-Lorenzo C., Concheiro A. (2006). Molecularly imprinted materials as advanced excipients for drug delivery systems. Biotechnology Annual Review.

[4] Miyata T. (2010). Preparation of smart soft materials using molecular complexes. Polym. J..

[5] Miyata T., Uragami T., Nakamae K. (2002). Biomolecule-sensitive hydrogels. Adv. Drug Deliv. Rev..

[6] Gong C., Wong K.L., Lam M.H.W. (2008). Photoresponsive molecularly imprinted hydrogels for the photoregulated release and uptake of pharmaceuticals in the aqueous media. Chem. Mater..

[7] Alvarez-Lorenzo C., Concheiro A. (2008). Intelligent drug delivery systems: Polymeric micelles and hydrogels. M. R. Med. Chem..

[8] Suedee R., Jantarat C., Lindner W., Viernstein H., Songkro S., Srichana T. (2010). Development of a pH-responsive drug delivery system for enantioselective-controlled delivery of racemic drugs. J. Control. Release.

[9] Miyata T., Jige M., Nakaminami T., Uragami T. (2006). Tumor marker-responsive behavior of gels prepared by biomolecular imprinting. Proc. Natl. Acad. Sci. USA.

[10] Cirillo G., Curcio M., Parisi O.I., Puoci F. (2010). Gastro-intestinal sustained release of phytic acid by molecularly imprinted microparticles. Pharm. Dev. Tech..

[11] Kryscio D.R., Peppas N.A. (2009). Mimicking biological delivery through feed back-controlled drug release systems based on molecular imprinting. AICHE J..

[12] Pande V.S., Grosberg A.Yu., Tanaka T. (1994). Thermodynamic procedure to synthesize heteropolymers that can renature to recognize a given target molecule. Proc. Natl. Acad. Sci. USA.

[13] Alvarez-Lorenzo C., Concheiro A. (2004). Molecularly imprinted polymers for drug delivery. J. Chromatogr. B.

[14] Alvarez-Lorenzo C., Concheiro A., Chuang J., Grosberg A.Yu., Galaev I., Mattiasson B. (2008). Imprinting using smart polymers. Smart Polymers, Applications in Biotechnology and Biomedicine.

[15] Puoci F., Iemma F., Muzzalupo R., Spizzirri U.G., Trombino S., Cassano R., Picci N. (2004). Spherical molecularly imprinted polymers (SMIPs) via a novel precipitation polymerization in the controlled delivery of salazine. Macromol. Biosci..

[16] Liu X.Y., Guan Y., Ding X.B., Peng Y.X., Long X.P., Wang X.C., Chang K. (2004). Design of temperature sensitive imprinted polymer hydrogels based on multiple-point hydrogen bonding. Macromol. Biosci..

[17] Demirel G., Ozcetin G., Turan E., Caykara T. (2005). pH/temperature-sensitive imprinted ionic poly (*N*-tert-butylacrylamide-co-acrylamide/maleic acid) hydrogels for bovine serum albumin. Macromol. Biosci..

[18] Burova T.V., Grinberg N.V., Kalinina E.V., Ivanov R.V., Lozinsky V.I., Alvarez-Lorenzo C., Grinberg V.Y. (2011). Thermoresponsive copolymer cryogel possessing molecular memory: Synthesis, energetics of collapse and interaction with ligands. Macromol. Chem. Phys..

[19] Fang L., Chen S., Zhang Y., Zhang H. (2011). Azobenzene-containing molecularly imprinted polymer microspheres with photoresponsive template binding properties. J. Mater. Chem..

[20] Kleinen J., Richtering W. (2011). Polyelectrolyte microgels based on poly-N-isopropylacrylamide: Influence of charge density on microgel properties, binding of poly-diallyldimethylammonium chloride, and properties of polyelectrolyte complexes. Colloid Polym. Sci..

[21] Fomenko A., Sedlakova Z., Ilavsky M. (2001). Phase transition in swollen gels—30. Temperature-induced phase transition in positively charged poly(N-isopropylacrylamide) hydrogels in water and aqueous NaCl solutions. Polym. Bull..

[22] Alvarez-Lorenzo C., Concheiro A., Dubovik A.S., Grinberg N.V., Burova T.V., Grinberg V.Y. (2005). Temperature-sensitive chitosan-poly(N-isopropylacrylamide) interpenetrated networks with enhanced loading capacity and controlled release properties. J. Control. Release.

[23] Alvarez-Lorenzo C., Concheiro A. (2002). Reversible adsorption by a pH- and temperature-sensitive acrylic hydrogel. J. Control. Release.

[24] Alvarez-Lorenzo C., Hiratani H., Tanaka K., Stancil K., Grosberg A.Yu., Tanaka T. (2001). Simultaneous multiple-point adsorption of aluminum ions and charged molecules by a polyampholyte thermosensitive gel: controlling frustrations in a heteropolymer gel. Langmuir.

[25] Grinberg V.Ya., Dubovik A.S., Kuznetsov D.V., Grinberg N.V., Grosberg A.Yu., Tanaka T. (2000). Studies of the thermal volume transition of poly(N-isopropylacrylamide) hydrogels by high-sensitivity differential scanning microcalorimetry. 2. Thermodynamic functions. Macromolecules.

[26] Burova T.V., Grinberg N.V., Dubovik A.S., Tanaka K., Grinberg V.Ya., Grosberg A.Yu. (2003). Effects of ligand binding on relative stability of subchain conformations of weakly charged N-isopropyl acrylamide gels in swollen and shrunken status. Macromolecules.

[27] Asoh T., Kaneko T., Matsusaki M., Akashi M. (2006). Rapid and precise release from nano-tracted poly(*N*-isopropylacrylamide) hydrogels containing linear poly (acrylic acid). Macromol. Biosci..

[28] Andrade-Vivero P., Fernández-Gabriel E., Alvarez-Lorenzo C., Concheiro A. (2007). Improving the loading and release of NSAIDs from pHEMA hydrogels by copolymerization with functionalized monomers. J. Pharm. Sci..

[29] Tanford C. (1968). Titration curves of proteins. Adv. Protein Chem..

[30] Woodbury C.P. (1993). The titration curve of weak polyacids. J. Phys. Chem..

[31] Rodriguez R., Alvarez-Lorenzo C., Concheiro A. (2003). Interactions of ibuprofen with cationic polysaccharides in aqueous dispersions and hydrogels. Rheological and diffusional implications. Eur. J. Pharm. Sci..

[32] Alvarez-Lorenzo C., Yañez F., Barreiro-Iglesias R., Concheiro A. (2006). Imprinted soft contact lenses as norfloxacin delivery systems. J. Control. Release.

[33] Chan A.W., Neufeld R.J. (2009). Modeling the controllable pH-responsive swelling and pore size of networked alginate based biomaterials. Biomaterials.

